# Description of recurrent headaches in 7–14-year-old children: Baseline data from a randomized clinical trial on effectiveness of chiropractic spinal manipulation in children with recurrent headaches

**DOI:** 10.1186/s12998-023-00479-z

**Published:** 2023-01-30

**Authors:** Kristina Boe Dissing, Werner Vach, Susanne Lynge, Henrik Wulff Christensen, Lise Hestbaek

**Affiliations:** 1 Chiropractic Knowledge Hub, Campusvej 55, 5230 Odense M, Denmark; 2 Basel Academy, Spalenring 145, 4055 Basel, Switzerland; 3 Private Practice, Vivaldisvej 6, 9700 Brønderslev, Denmark; 4 University of Southern Denmark, Campusvej 55, 5230 Odense M, Denmark

**Keywords:** Headache, Children, Classification, Diagnosis

## Abstract

**Background:**

Headaches in children are poorly described and diagnosing can be challenging. Objectives are: (1) to describe headache characteristics and child characteristics, (2) to explore whether data can suggest a more diverse way to categorize headaches than traditionally.

**Methods:**

Baseline data for a clinical trial included a questionnaire and a physical screening. Children's characteristics and detailed description of headache symptoms were provided. Children were classified for migraine or tension-type-headache based on questionnaire data reported by children and parents. This required to apply slightly modified classification criteria and a “non-classifiable” group was added. Severity and symptoms, related to the migraine versus tension type distinction, were investigated to define a migraine-tension-type-index.

**Results:**

253 children were included. Mean pain intensity was 5.9/10. Over 2/3 of the children had headache for > 1 year, and > 50% for several days/week. Half of the children were non-classifiable, 22% were classified as migraine and 23% as tension-type headache. A migraine-tension-type-index was constructed and describes a continuous spectrum rather than two distinct groups.

**Conclusions:**

Children with recurrent headaches are often severely affected. A questionnaire-based classification appeared feasible to distinguish between migraine and tension-type headaches in children but leaving many children unclassified. A migraine-tension-type-index can be generated allowing to regard the traditional distinction as a continuum (including mixed headache), and potentially serving as an instrument to improve headache management.

*Trial registration* ClinicalTrials.gov, identifier NCT02684916.

## Introduction

Headache in children is common, often recurring, and prevalence rises from 5% at the age of three to more than 50% in puberty [1, 2]. Recurrent headaches may impact many aspects of childhood, such as quality of life and school performance [3, 4], social life [3], mental health [5, 6], and participation in physical activities [7]. Furthermore, children with recurrent headaches suffer from more comorbid physical conditions, such as abdominal pain, neck and back pain, overweight, sleep problems, and allergies [5, 8–12]. This emphasizes the need for early identification and proper management of children with recurrent headache, to minimize the risk of long-term consequences, not least because children experiencing recurrent headaches also have a higher risk of suffering from headaches in adulthood [13, 14].

Basic knowledge of headache characteristics and demographic factors is important to aid proper management and prevention. Treatment strategies for headaches are typically founded on the diagnosis of headache type, based on the International Classification of Headache Disorders (ICHD) [15]. It can be challenging to categorize younger children with headaches, because they may have difficulties describing their symptoms, especially regarding pain quality, pain score and presence of pain-aggravating factors [16, 17]. Furthermore, it seems that children often are unclassifiable or have coexisting headaches, primarily migraine and tension-type headache [16], which are the most commonly recurring headaches in children [18, 19]. It would be desirable for both clinicians and researchers to take this uncertainty into account. Regarding the distinction as the end points of a continuum may be one way to approach this.

To improve our knowledge about children with recurrent headaches we will take advantage of a population sampled for a randomized controlled trial (RCT) investigating the effectiveness of chiropractic spinal manipulation in children aged 7–14 with recurrent headaches [20, 21]. This study collected extensive baseline information on more than 200 children and therefore a detailed description of these children will be reported regarding headache characteristics, co-morbidity, trauma, and physical activity. Thus, the objectives of this study are (1) to describe headache characteristics and child characteristics in children with recurrent headaches, (2) to explore whether data can suggest a more diverse way to categorize headaches than traditionally, by generating a new index.

## Materials and methods

### Setting

Data were collected at two clinics (one chiropractic clinic and one pediatric specialty practice) in Northern Denmark. Screenings were performed by the same investigating chiropractor. All details can be seen in the study protocol of the RCT [20].

### Participants

From November 2015 to August 2019, children aged 7–14 years with recurrent headaches were recruited to the trial through the Danish School Information Network, local newspapers, television, social media, and radio. Participants were included in the trial if they (1) were between 7–14 years old, (2) had experienced at least one episode of headache per week for the past 6 months, and (3) had at least one musculoskeletal dysfunction in the spine, pelvis or temporomandibular joints found at the physical screening. Before the screening for the trial, all eligible children and their parents answered three questions each week for four weeks via a text message system on their mobile (number of days with a headache, intensity of headaches and number of pills taken for headache during the previous week), thereby verifying having at least one episode of headache per week for four weeks. In addition, a baseline questionnaire was completed.

### Variables

Data for this study were based on the baseline questionnaire (“Appendix 1”) as well as clinical data from the physical screening. The questionnaire was based on relevant literature and knowledge obtained from a non-published scoping review by the primary investigator, as part of her dissertation for a master’s degree in Musculoskeletal Pediatrics. It was completed by the parents/caregiver and the child. All children with data from both the baseline questionnaire and the screening were included, regardless of participation or non-participation in the subsequent trial. All included variables in this study are described in detail in “Appendix 2”, including specification of content, response format and source.

### Classification of headaches

To develop the index, we needed to categorize the children into having migraine or tension-type headaches. The standard method when diagnosing headaches are using interviews and headache diaries, and optionally validated questionnaires. The children evaluated for participation in the trial were included due to experiencing recurrent headaches and not by diagnosis, and therefore the available data does not cover the ICHD criteria entirely, so the categorization had to be slightly modified (Table 1). Criteria A was considered to be fulfilled for both migraine and tension-type headaches since the children were having weekly headache. A migraine diagnosis was allocated if criteria B-D were fulfilled. For tension-type headaches, we ignored information on duration since the response options were not detailed enough. Hence a diagnosis was allocated if criteria C + D was fulfilled. We chose not to distinguish between migraine with or without aura symptoms and used the criteria for migraine without aura. Children not fulfilling the criteria for migraine or tension-type-headache were regarded as a third group labeled as having “non-classifiable headache”.

**Table 1:** Headache diagnoses by available data according to the International Classification of Headache Disorders (ICHD)

ICHD classification criteria	Available data
*Migraine*	
A. At least five attacks fulfilling criteria B–D	Since all the children had weekly headaches, we assumed the criterion of five attacks to be fulfilled for everybody
B. Headache attacks lasting 2–72 h	Our data contains one corresponding item with the answer options: “Less than 2 h”, “2 h up to half a day”, “The whole day”, “All day and all night”. If one of the three latter was chosen, we considered the criterion to be fulfilled
C. Headache has at least two of the following four characteristics:	Headache has at least two of the following three available characteristics:
1. Unilateral location	The variable “location” did not directly include a distinction between unilateral and bilateral location. Migraine in children under 18 years are often bilateral. However, we chose to explore if the definition for adults was suitable for children and therefore regarded the criterion as fulfilled, if the answer option “one side of the head” or “behind one eye” was chosen
2. Pulsating quality	Unfortunately, we did not have a variable on the quality of the headache. Hence, this criterion was ignored
3. Moderate to severe pain intensity	The children rated their pain intensity on the numerical rating scale from 0 to 10. This was then categorized into mild (0–4), moderate (5–6) and severe pain (7–10)
4. Aggravation by or causing avoidance of routine physical activity (e.g. walking or climbing stairs)	The only relevant variable for this criterion was “Aggravated by sports”*.* The criterion was assumed to be fulfilled if the answer was “yes”
D. During headache at least one of the following:	
1. Nausea and/or vomiting	Corresponding items were available and used
2. Photophobia and phonophobia	Corresponding items were available and used
*Frequent episodic tension-type headache*	
A. At least 10 episodes of headache occurring 1–14 days/month on average for > 3 months (≥ 12 and < 180 days/year) and fulfilling criteria B–D	The children were having weekly headaches hence we assumed this criterion to be fulfilled
B. Lasting from 30 min to seven days	The response options for the question on the duration distinguished only between ‘less than 2 h’ and ‘above 2 h’. Hence, less than 30 min was not an option, and this criterion was ignored
C. At least two of the following four characteristics:	Headache has at least two of the following three available characteristics:
1. Bilateral location	We did not have a location named ‘bilateral’, but we regarded the criterion as fulfilled if the answer option named ‘the whole head’ was used
2. Pressing or tightening (non-pulsating) quality	Unfortunately, we did not have a variable on the quality of the headache. Hence this criterion was ignored
3. Mild or moderate intensity	The children rated their pain intensity on the numerical rating scale from 0 to 10. This was then categorized into mild (0–4), moderate (5–6) and severe pain (7–10)
4. Not aggravated by routine physical activity such as walking or climbing stairs	The criterion was assumed to be fulfilled if the answer to “Aggravated by sports” was “no”
D. Both of the following:	
1. No nausea or vomiting	Corresponding items were available and used
2. No more than one of photophobia or phonophobia	Corresponding items were available and used

### Statistical analyses

The child- and headache characteristics are described by absolute and relative frequencies and means with 10th and 90th percentiles for all children and stratified by headache type. The statistical significance of differences between the three groups were assessed using the Kruskal Wallis test for continuous and ordinal variables, and an extension of Fisher’s exact test for binary and categorical variables. Kernel density estimates were used to depict distributional differences in continuous variables between subgroups of children.

### Construction of a migraine-tension-type-index

Instead of applying an existing categorization, it is possible to use our data to investigate whether the distinction between migraine and tension-type headache also makes sense in children, and to explore whether our data can suggest a more diverse way to categorize headaches than the traditional categorization. If there is evidence for this, it might be possible to develop an index helping to identify those children who can be assigned definitely to one type and to evaluate the degree of either migraine or tension type headache in children with mixed headaches.

According to the ICHD, migraine and tension-type-headache differ with respect to severity, co-occurring symptoms (nausea/vomiting, photophobia and phonophobia), relation to routine physical activity, and location. As pointed out above, closely corresponding information was available in our data. Consequently, we explored the correlation among these variables to check, whether corresponding dimensions were identifiable and tried to combine the emerging dimensions into one index. We approached this step by inspection of association measures (Pearson correlation, Odds ratios) for each pair of variables. We used a factor analysis with varimax rotation to identify latent variables/dimensions. To summarize the information from several variables into one index, the first principal component score was used. In this score, the weights of each variable are chosen in the way maximizing the variance of the resulting score.

The following variables were included in this analysis:

- Aspects of severity: Intensity, frequency, length of headache episode, duration of headache prior to entering the project, the use of medicine and number of days absent from school.
- Co-occurring symptoms: nausea, vomiting, phonophobia, photophobia
- Aggravation: Physical activity
- Location of headache

### Extending the categorization to include a “probable” state

The ICHD classification allows to define also probable migraine and probable tension-type headaches in those children fulfilling only 3 of the 4 criteria. Following this idea, we extended our classification system accordingly. “Probable migraine” was considered if 2 out of 3 criteria B-D were fulfilled, and “probable tension-type headache” was considered if 1 out of 2 criteria C + D was fulfilled.

## Results

### Description of children with recurrent headaches

There were 283 children eligible for the pre-screening data collection period, 30 children did not fulfill the inclusion criteria for the screening process and were therefore excluded, and hence, the baseline cohort consisted of 253 children, with 44% boys and a median age of 11 [21]. Descriptive information about the cohort can be found in the “total” column of Tables 2 and 3.

**Table 2:** Headache characteristics used to categorize the headache diagnoses reported by headache type

	Tension-type	Migraine	Non-classifiable	Total
n (%)	58 (22.9)	56 (22.1)	139 (54.9)	253 (100.0)
*Frequency of headache, n (%)*				
1–2 days a week	19 (32.8)	22 (39.3)	66 (47.5)	107 (42.3)
3–5 days a week	26 (44.8)	25 (44.6)	60 (43.2)	111 (43.9)
Almost every day	13 (22.4)	9 (16.1)	13 (9.4)	35 (13.8)
*Duration of episode, n (%)*				
Less than 2 h	8 (13.8)	0 (0.0)	21 (15.1)	29 (11.5)
2 h to 1/2 day	38 (65.5)	42 (75.0)	91 (65.5)	171 (67.6)
Whole day	12 (20.7)	12 (21.4)	23 (16.5)	47 (18.6)
All day and night	0 (0.0)	2 (3.6)	4 (2.9)	6 (2.4)
*Typical location of headache, n (%)*				
Whole head	11 (19.0)	2 (3.6)	23 (16.5)	36 (14.2)
Backside of head	4 (6.9)	3 (5.4)	2 (1.4)	9 (3.6)
One side of head	6 (10.3)	14 (25.0)	7 (5.0)	27 (10.7)
Neck	0 (0.0)	3 (5.4)	3 (2.2)	6 (2.4)
Forehead	21 (36.2)	9 (16.1)	58 (41.7)	88 (34.8)
Behind one eye	2 (3.4)	10 (17.9)	3 (2.2)	15 (5.9)
Varies a lot	14 (24.1)	15 (26.8)	43 (30.9)	72 (28.5)
*Co-occurring symptoms*				
Nausea, n (%)	0 (0.0)	45 (80.4)	89 (64.0)	134 (53.0)
Vomit, n (%)	0 (0.0)	18 (32.1)	36 (25.9)	54 (21.3)
Photophobia, n (%)	11 (19.0)	44 (78.6)	84 (60.4)	139 (54.9)
Phonophobia, n (%)	18 (31.0)	48 (85.7)	92 (66.2)	158 (62.5)
Aggravated by sports, n (%)	0 (0.0)	42 (75.0)	26 (18.7)	185 (73.1)
Typical pain intensity (NRS*), mean (95% CI**)	4.8 (4.5; 5.2)	6.6 (6.3; 6.9)	6.1 (5.9; 6.4)	5.9 (5.7; 6.1)

*NRS numerical rating scale, **CI confidence interval

**Table 3:** Baseline information by headache category

	Tensiontype	Migraine	Non-classifiable	Total	*p* Value
n (%)	58 (22.9)	56 (22.1)	139 (54.9)	253 (100.0)	
Age, median (p10; p90)	11.0 (8;14)	11.0 (8;13)	11.0 (8;13)	11.0 (8;14)	0.39
Age boys, median (p10; p90)	11.0 (8;14)	12.0 (8;13)	11.0 (8;14)	11.0 (8;14)	0.52
Age girls, median (p10; p90)	11.0 (7;14)	11.0 (8;14)	11.0 (7;13)	11.0 (7;14)	0.51
*Sex, n (%)*					0.90
Boy	27 (46.6)	25 (44.6)	60 (43.2)	112 (44.3)	
Girl	31 (53.4)	31 (55.4)	79 (56.8)	141 (55.7)	
Height, median (p10; p90)	152.5 (134;168)	149.5 (138;166)	149.0 (131;170)	150.0 (134;168)	0.39
Weight, median (p10; p90)	45.0 (26.9;64.2)	40.0 (30.5;58.2)	38.5 (26.6;58.9)	39.4 (27.2;59.5)	0.40
Overweight, n (%)	12 (20.7)	9 (16.1)	20 (14.4)	41 (16.2)	0.55
Obese, n (%)	1 (1.7)	1 (1.8)	8 (5.8)	10 (4.0)	0.38
Cervical dysfunction* n (%)	40 (69.0)	41 (73.2)	108 (77.7)	189 (74.7)	0.41
Decreased cervical ROM**, n (%)	45 (77.6)	45 (80.4)	118 (84.9)	208 (82.2)	0.41
Thoracic dysfunction*, n (%)	38 (65.5)	42 (75.0)	110 (79.1)	190 (75.1)	0.13
Lumbar dysfunction*, n (%)	20 (34.5)	29 (51.8)	55 (39.6)	104 (41.1)	0.14
Pelvic dysfunction*, n (%)	21 (36.2)	21 (37.5)	58 (41.7)	100 (39.5)	0.75
TMJ dysfunction*, n (%)	9 (15.5)	11 (19.6)	32 (23.0)	52 (20.6)	0.50
*Duration of headache, n (%)*					0.06
1/2–1 year	19 (32.8)	8 (14.3)	27 (19.4)	54 (21.3)	
1–3 years	29 (50.0)	27 (48.2)	71 (51.1)	127 (50.2)	
More than 3 years	10 (17.2)	21 (37.5)	41 (29.5)	72 (28.5)	
*Typical onset of attack, n (%)*					0.07
Morning	3 (5.2)	3 (5.4)	22 (15.8)	28 (11.1)	
Late morning	16 (27.6)	7 (12.5)	24 (17.3)	47 (18.6)	
Afternoon	12 (20.7)	14 (25.0)	36 (25.9)	62 (24.5)	
Evening/night	0 (0.0)	1 (1.8)	4 (2.9)	5 (2.0)	
Varies day and night	27 (46.6)	31 (55.4)	53 (38.1)	111 (43.9)	
*Co-occurring symptoms with headache*					
Dizziness, n (%)	22 (37.9)	32 (57.1)	76 (54.7)	130 (51.4)	0.07
Stomach pain, n (%)	11 (19.0)	23 (41.1)	46 (33.1)	80 (31.6)	0.03
Visual disorder, n (%)	15 (25.9)	19 (33.9)	31 (22.3)	65 (25.7)	0.25
Spots in front of eyes, n (%)	9 (15.5)	16 (28.6)	27 (19.4)	52 (20.6)	0.21
Numbness arms, n (%)	4 (6.9)	5 (8.9)	8 (5.8)	17 (6.7)	0.66
Other symptoms, n (%)	3 (5.2)	3 (5.4)	15 (10.8)	21 (8.3)	0.36
*Trigger factors*					
Neck pain, n (%)	25 (43.1)	28 (50.0)	64 (46.0)	117 (46.2)	0.75
Back pain, n (%)	14 (24.1)	14 (25.0)	28 (20.1)	56 (22.1)	0.68
Stress, n (%)	18 (31.0)	26 (46.4)	61 (43.9)	105 (41.5)	0.17
Sitting, n (%)	17 (29.3)	22 (39.3)	56 (40.3)	95 (37.5)	0.35
Reading, n (%)	20 (34.5)	26 (46.4)	43 (30.9)	89 (35.2)	0.13
Computer/tv, n (%)	25 (43.1)	31 (55.4)	70 (50.4)	126 (49.8)	0.43
Menstruation, n (%)	3 (5.2)	0 (0.0)	8 (5.8)	11 (4.3)	0.18
*Relieving factors*					
Lying down, n (%)	27 (46.6)	35 (62.5)	78 (56.1)	140 (55.3)	0.23
Sleeping, n (%)	42 (72.4)	48 (85.7)	116 (83.5)	206 (81.4)	0.13
Eating, n (%)	11 (19.0)	17 (30.4)	41 (29.5)	69 (27.3)	0.27
Something to drink, n (%)	30 (51.7)	32 (57.1)	77 (55.4)	139 (54.9)	0.83
Fresh air, n (%)	26 (44.8)	27 (48.2)	67 (48.2)	120 (47.4)	0.91
Sports, n (%)	8 (13.8)	4 (7.1)	22 (15.8)	34 (13.4)	0.27
Medicine, n (%)	40 (69.0)	45 (80.4)	100 (71.9)	185 (73.1)	0.35
Neck pain last year, n (%)	32 (55.2)	30 (53.6)	89 (64.0)	151 (59.7)	0.29
Back pain last year, n (%)	22 (37.9)	28 (50.0)	57 (41.0)	107 (42.3)	0.39
Braces, n (%)	7 (12.1)	5 (8.9)	9 (6.5)	21 (8.3)	0.41
*Non-prescriptive medicine, n (%)*					0.33
Never	9 (15.5)	4 (7.1)	16 (11.5)	29 (11.5)	
1–3 Times per month	36 (62.1)	28 (50.0)	76 (54.7)	140 (55.3)	
1–3 Times per week	12 (20.7)	20 (35.7)	42 (30.2)	74 (29.2)	
More than 3 times per week	1 (1.7)	4 (7.1)	5 (3.6)	10 (4.0)	
Prescriptive medicine, n (%)	0 (0.0)	5 (8.9)	5 (3.6)	10 (4.0)	0.05
Medication other disease, n (%)	6 (10.3)	8 (14.3)	12 (8.6)	26 (10.3)	0.46
Examination general practitioner, n (%)	20 (34.5)	38 (67.9)	80 (57.6)	138 (54.5)	0.00
Examination paediatric, n (%)	5 (8.6)	17 (30.4)	34 (24.5)	56 (22.1)	0.01
Previous treatment, n (%)	13 (22.4)	13 (23.2)	24 (17.3)	50 (19.8)	0.51
General practitioner, n (%)	7 (53.8)	6 (46.2)	5 (20.8)	18 (36.0)	0.09
Paediatrician, n (%)	2 (15.4)	8 (61.5)	8 (33.3)	18 (36.0)	0.06
Physiotherapist, n (%)	5 (38.5)	5 (38.5)	9 (37.5)	19 (38.0)	1.00
Chiropractor, n (%)	6 (46.2)	8 (61.5)	11 (45.8)	25 (50.0)	0.76
Other***, n (%)	8 (61.5)	8 (61.5)	13 (54.2)	29 (58.0)	0.87
*Trauma to head/neck without need to see doctor, n (%)*					0.74
0 times	23 (39.7)	16 (28.6)	49 (35.3)	88 (34.8)	
1–3 times	24 (41.4)	25 (44.6)	61 (43.9)	110 (43.5)	
More than 3 times	11 (19.0)	15 (26.8)	29 (20.9)	55 (21.7)	
*Trauma to head/neck with need to see doctor, n (%)*					0.08
0 times	42 (72.4)	33 (58.9)	95 (68.3)	170 (67.2)	
1–3 times	13 (22.4)	18 (32.1)	42 (30.2)	73 (28.9)	
More than 3 times	3 (5.2)	5 (8.9)	2 (1.4)	10 (4.0)	
Concussion, n (%)	9 (15.5)	16 (28.6)	28 (20.1)	53 (20.9)	0.23
Allergies, n (%)	20 (34.5)	21 (37.5)	40 (28.8)	81 (32.0)	0.44
*Stomach pain, n (%)*					0.50
No	12 (20.7)	6 (10.7)	28 (20.1)	46 (18.2)	
1–5 times a year	19 (32.8)	20 (35.7)	41 (29.5)	80 (31.6)	
6–12 times a year	11 (19.0)	16 (28.6)	26 (18.7)	53 (20.9)	
More than once a month	16 (27.6)	14 (25.0)	44 (31.7)	74 (29.2)	
Smoking in the home, n (%)	9 (15.5)	11 (19.6)	22 (15.8)	42 (16.6)	0.76
*Number of days with sport per week, n (%)*					0.05
0 times	9 (15.5)	6 (10.7)	5 (3.6)	20 (7.9)	
1–3 times	39 (67.2)	37 (66.1)	101 (72.7)	177 (70.0)	
More than 3 times	10 (17.2)	13 (23.2)	33 (23.7)	56 (22.1)	
*Screen time per day, n (%)*					0.33
0–1 h	7 (12.1)	4 (7.1)	10 (7.2)	21 (8.3)	
2–4 h	35 (60.3)	37 (66.1)	102 (73.4)	174 (68.8)	
5–6 h	12 (20.7)	8 (14.3)	14 (10.1)	34 (13.4)	
More than 6 h	4 (6.9)	7 (12.5)	13 (9.4)	24 (9.5)	
Poor sleep, n (%)	6 (10.3)	6 (10.7)	8 (5.8)	20 (7.9)	0.31
*Menstruation, (N* = *141), n (%)*					0.75
No	21 (67.7)	24 (77.4)	62 (78.5)	107 (75.9)	
Yes, start 12–14 years of age	5 (16.1)	4 (12.9)	10 (12.7)	19 (13.5)	
Yes, start 8–11 years of age	5 (16.1)	3 (9.7)	7 (8.9)	15 (10.6)	
Glasses/contact lenses, n (%)	12 (20.7)	7 (12.5)	18 (12.9)	37 (14.6)	0.35

*Dysfunction is defined as a biomechanical dysfunction resulting in reduced ranges of motion of one or more joints found during clinical examination of the child’s spine, pelvis or temporomandibular joint **ROM: range of motion ***Other: reflexology, massage, other

The mean pain intensity at baseline was 5.9 measured on a numerical rating scale. More than 2/3 of the children had been suffering from headache for more than a year and more than 50% for several days a week. The duration of attacks varied, but most commonly they lasted from two to twelve hours per day*.*

More than 90% of the children were engaged in sports one or more times per week, screen time per day was most commonly two to four hours, and more than 90% slept well. All children experienced some co-occurring symptoms, most often nausea, dizziness, phonophobia or photophobia. The most frequent triggering factors of headache were sports and use of computer/tv, and the most frequent relieving factors were sleep and medicine. Cervical dysfunctions and decreased cervical range of motion was found in 3/4 of the children, and 60% had experienced neck pain within the last year. Around 2/3 of the children had experienced trauma to the head and/or neck without seeing a doctor or going to the hospital, and 1/3 had experienced trauma needing medical attention.

More than half of the children had been examined by a general practitioner and 20% had received treatment for headache before entering the study. More than 1/3 of the children used non-prescriptive medicine one or more days a week.

### Differences between children classified with migraine, tension-type headache, or non-classifiable headache

Half of the children did not fall into one of the two diagnostic types, 22% of the children were categorized with migraine and 23% with tension-type headache. Table 2 depicts the differences in headache characteristics defining the diagnoses. The differences between tension-type headache and migraine were most pronounced with respect to co-occurring symptoms and aggravation by sports.

Non-classifiable children tend to have headache less frequently, a lower episode duration and more varying pain or pain in the forehead.

Table 3 shows the differences between diagnostic groups for variables not used as diagnostic criteria. For most variables no distinct differences could be observed. However, in the tension-type group the duration before screening was shorter, children with migraine took more medication and consulted health care professionals more often (both for examination and treatment). Only few of these differences were statistically significant.

#### Construction of a migraine-tension-type-index

For the six variables related to severity, we observed only very moderate correlations (“Appendix 3”). Interestingly, the number of sick days was correlated with all aspects, suggesting that this variable might be seen as a summary measure.

A factor analysis suggested to reduce the severity variables to two factors: one factor related to all variables except frequency, and another factor related to frequency and length of episode (“Appendix 4”).

**Fig. 1:**
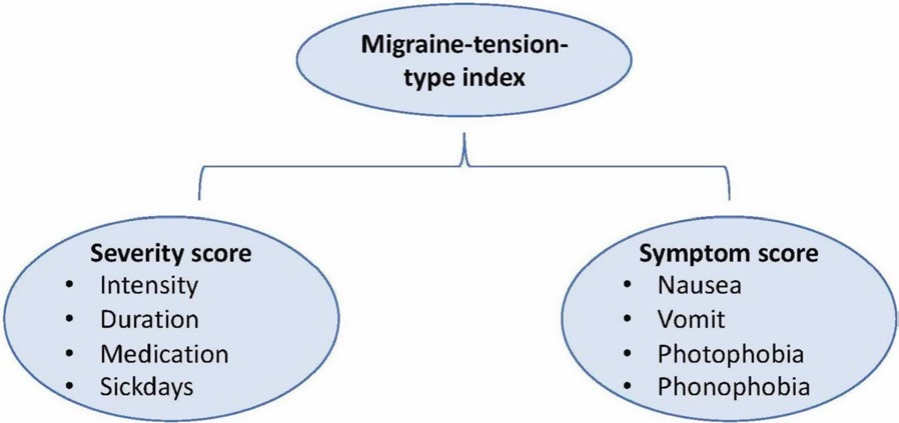
Migraine-tension-type index. Footnote: The severity score was computed as 0.527 * intensity + 0.477 * duration + 0.433 * medication + 0.554 * sick days after rescaling the single variables to mean 0 and standard deviation 1

This is in line with the distinction between migraine and tension-type headache, where intensity plays a major role, whereas the role of frequency is less clear. To compute the first summary variable (severity score), we applied a principal component analysis (PCA) based on the severity variables except frequency and length of episode (i.e. intensity, duration of headache prior to entering the project, the use of medicine, and the number of days absent from school), and obtained the weights indicated in Fig. 1.

The four co-occurring symptoms nausea, vomiting, phonophobia and photophobia tended to be rather distinctly associated (ORs between 1.8 and 43.2, see “Appendix 5”) with the highest association between nausea and vomiting (OR = 43.2). We defined a symptom score by counting the presence of the single symptoms, resulting in a symptom score from 0 to 4 (Fig. 1).

We observed a distinct association between the severity score and the symptom score (r = 0.45), which supports the assumption that the distinction between migraine and tension-type headache using severity and co-occurring symptoms is as reasonable in children as in adults. We therefore defined a migraine-tension-type-index as a PCA-based summary score based on both the severity and the symptom scores.

**Fig. 2:**
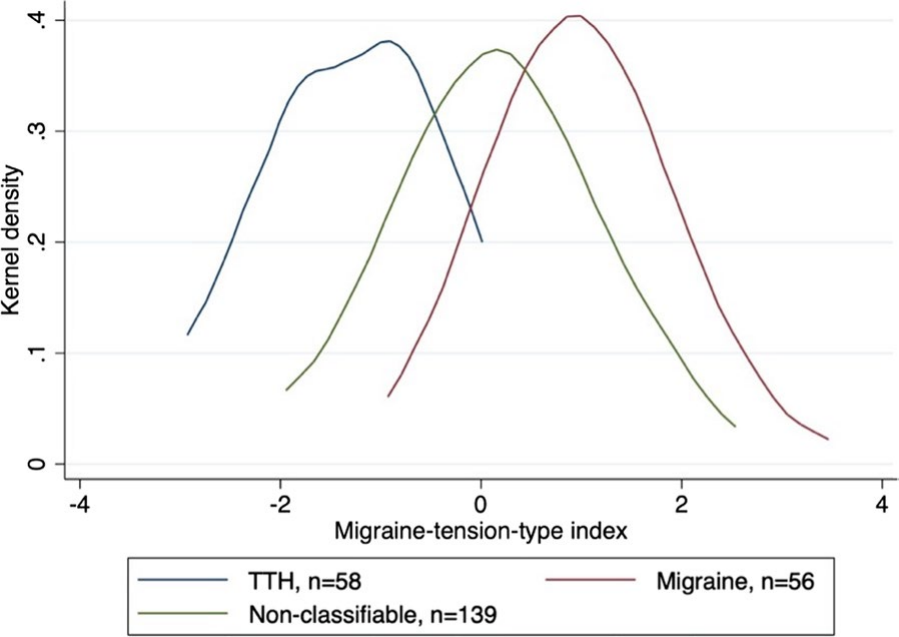
Migraine-tension-type index distribution in diagnostic subgroups

This new index describes a continuous spectrum and not just two distinct groups, but probably with migraine in the high end of the index and tension-type headache in the low end. This is supported by the distribution of the index in the three diagnostic subgroups (Fig. 2): Children classified as migraine and tension-type headache, respectively, define two rather well separated groups.

Interestingly, the non-classifiable children do not just constitute a distinct in-between-group, but partially include children with rather low or high index values.

One advantage of this index is the possibility to include all children in investigations of the relation between the migraine-tension-type distinction and other variables.

**Fig. 3:**
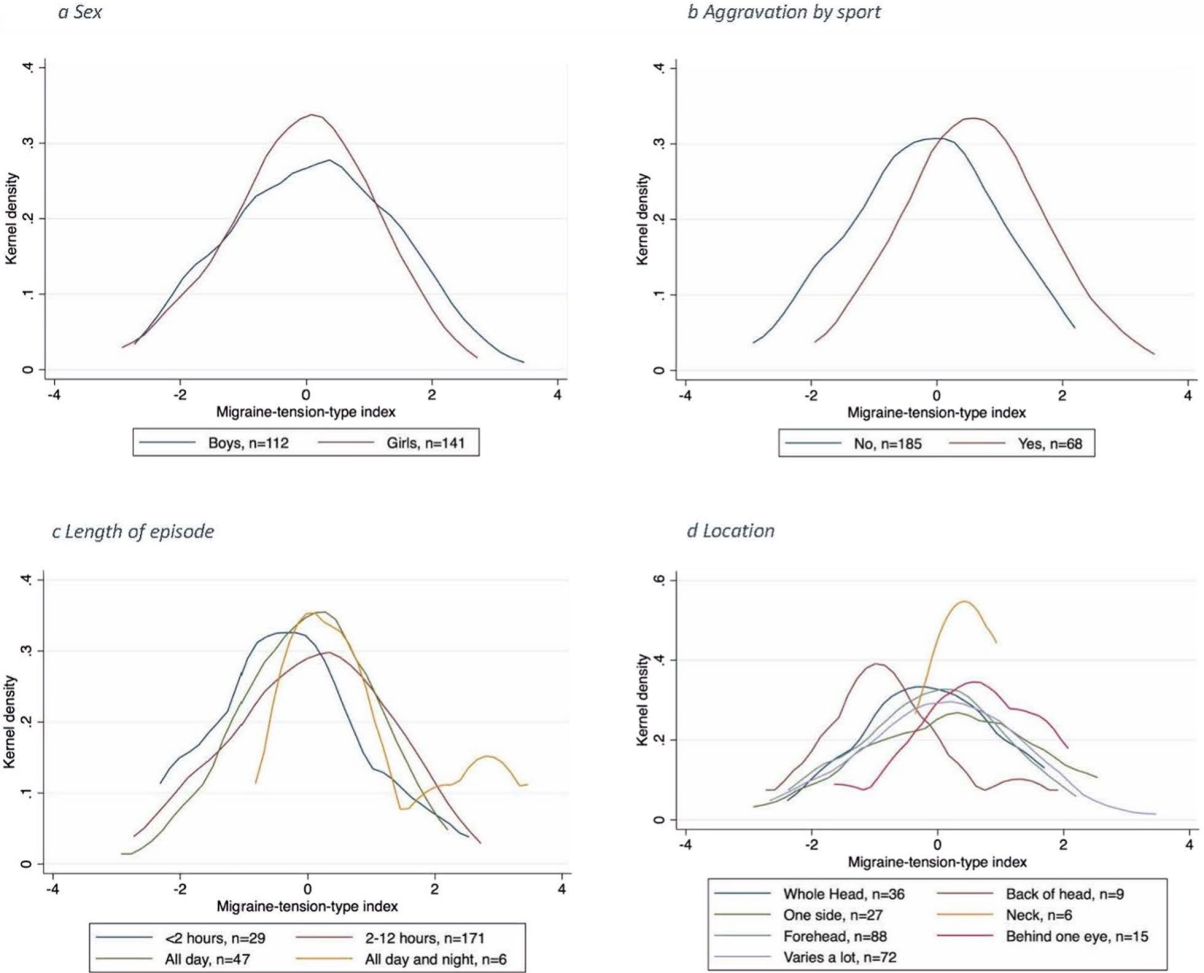
Migraine tension-type headache index associations with headache characteristics

Figure 3a depicts the distribution of the index within boys and girls, and we can observe that girls have a rather symmetric distribution, whereas in boys there is a slightly skewed distribution with a tendency to more extreme values in the direction of migraine. Furthermore, we investigated the relation of the migraine-tension-type distinction to the classification variables not included in the index; that is length of episode, aggravation by sports and location of headache (Fig. 3b–d). Figure 3b depicts that aggravation by sports is associated with index values in the direction of migraine which is in line with the diagnostic criteria. Regarding length of episode (Fig. 3c) there is a tendency towards low index values in the direction of tension-type headache for those with short-lasting episodes and for high values for those with long-lasting episodes. As for location, the most distinct differences seen in Fig. 3d are between ‘Back of head’ trending towards low values, i.e. tension-type headache, and ‘Behind one eye’ trending towards high values, i.e. migraine. The latter holds also for ‘Neck’, but these three groups include very few children and thus this should be interpreted with caution.

#### Extending the classification system

**Fig. 4:**
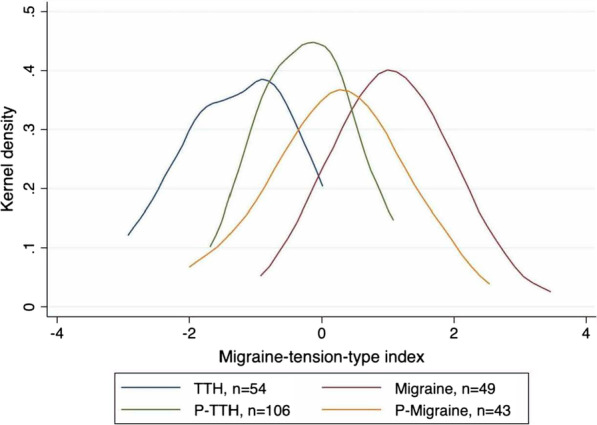
Migraine-tension-type index distribution in probable versus definite headache diagnoses. *TTH: tension-type headache, P-TTH: probable tension-type headache, P-Migraine: probable migraine. The groups “Migraine” and “TTTH” do only include those children, who are not simultaneously also in another class

Table 4 indicates the distribution of the number of criteria fulfilled by each child. It illustrates a basic limitation when trying to extend the classification system by allowing two additional “probable” states. Due to the low number of criteria available, a substantial number of children is now classified into two classes. Although this limits the practical usefulness of this extension, it allows to illustrate the ability of the new index to define a continuum. The four groups of children differ in the mean values of the index but are substantially overlapping as shown in Fig. 4.

**Table 4:** Combination of criteria (B-D) fulfilled for migraine and tension-type headaches

# of criteria fulfilled for migraine	Classification	# of criteria fulfilled for tension-type	Classification	Frequency	Percent
2	Probable migraine	1	Probable tension-type	78*	30.8*
3	Migraine	0		49	19.4
1		2	Tension type	47	18.6
2	Probable migraine	0		39	15.4
1		1	Probable tension-type	20	7.9
3	Migraine	1	Probable tension-type	7*	2.8
0		2	Tension type	7	2.8
2	Probable migraine	2	Tension type	4*	1.6
0		1	Probable tension-type	1	0.4
1		0		1	0.4

*Children classified with more than one type of headache

## Discussion

### The cohort

The cohort included more girls than boys which has also been reported elsewhere for children with recurrent headache [2, 22, 23]. The distribution of sex and age is the same across the three types of headaches. Most of the children have been suffering for headache for a long time before entering the study, especially children in the migraine group. This supports the importance of being alert to children with headaches early in their life, to minimize the risk of lifelong recurrent suffering.

Kroner-Herwig et al. [12] conducted a study on 7–14-year-old German children, and they provided a diagnosis on migraine, tension-type headache, and non-classifiable headache, based on questionnaire data, like we did. In contrast to our study, this was a population-based study and not restricted to a certain severity. However, the distribution of the co-occurring symptoms between the three groups were somewhat similar to ours but with more symptoms related to migraine and non-classifiable headache than to tension-type headache.

Most of the children in our study were physically active with less than 10% not participating in sports. However, those not participating in sports may do a lot of active leisure time activities, which are not considered ‘sports’ and therefore not captured by the questionnaire. Interestingly, children in the tension-type headache group were least physically active but had less severe pain than the other two groups. According to the ICHD, tension-type headache is usually not aggravated by physical activity in contrast to migraine, and hence the assumption would be, that children with migraine were the least active, also since they have the highest level of pain. This contradiction could indicate that pain is not the most bothersome issue for children with headache, maybe the quality of the headache has a higher impact on daily life, or maybe physical activity prevents or diminishes tension-type headache.

This cohort consists of children with recurrent or chronic headaches. Nevertheless, only half of the children had consulted a general practitioner due to their headache, which was also found in the study by Kroner-Herwig et al. [12]. In our study distinctly more children with migraine, (and to a lesser extent also non-classifiable headache) consulted a general practitioner or a pediatrician than children with tension-type headache. Interestingly, only 20% of the participants reported the child to have received treatment, but the medication rate was rather high with 90% of the children taking medicine, and more than 1/3 of the children using non-prescriptive medication more than once a week. The high medication use emphasizes the need for optimizing non-pharmacological means of preventing or treating headache, and to improve knowledge among both parents and healthcare practitioners. Overall, the frequencies with respect to the different variables related to seeking help indicate a great variation in whether and how parents seek help.

### Classification of headaches

There can be several explanations for the high number (55%) of non-classifiable children in our study. One explanation is the challenge of classifying headaches in children using the available questionnaire-based data. Some of the ICHD criteria could not be checked, and for some we only had limited degree of information, which could mean that some headaches may meet the criteria for “probable migraine” or “probable tension-type headache”. An attempt to extend our classification system in this direction failed, as too few criteria could be applied. Part of the explanation for the substantial number of unclassifiable children could be co-existing headache types (mixed headaches) making a specific diagnosis difficult due to overlapping symptoms [24, 25].However, other diagnoses might also be present in this patient population. One potential diagnosis could be medication overuse headache, which—considering the high use of non-prescriptive medication (more than 1/3 of the children in this cohort used non-prescriptive medicine one or more days a week, Table 3)—is highly likely. Another diagnosis could be cervicogenic headache. This might be an important diagnosis in this age group, where children are prone to frequent trauma. In the non-classifiable group, there were 20% with concussions, 64% with neck pain and 85% with decreased cervical ROM; the latter two frequencies were higher than the migraine and tension-type groups. According to the ICHD criteria, the cervicogenic headaches include several migrainous features (nausea, vomit, and photo/phonophobia), and for our cohort the co-occurring symptoms were generally more prevalent in the non-classifiable group than for tension-type headache. Unfortunately, we lack the necessary variables to define these two diagnoses in this study.

Furthermore, it can be challenging for parents and children to answer items related to pain (intensity, quality, and aggravation factors), which are highly relevant for distinguishing between migraine and tension-type headache [17]. In our study, the questionnaire was assumed to be filled out by the child and the parents together, but discrepancies in the grading of pain between a child and a parent have been reported elsewhere and is a common source of uncertainty in studies investigating paediatric pain [26–28], making the validity of the pain intensity variable uncertain.

#### The migraine-tension-type-index

An index based on a combination of severity and co-occurring symptoms appeared to be useful by allowing children to be placed on a continuum between tension-type headache and migraine rather that in dichotomized categories, thereby also taking mixed headaches into account. The index could indicate that the state of headache may be more fluent between diagnoses and not so distinctly defined.

Such an index could be advantageous as it allows for combination of headaches with overlapping symptoms, which is supported by the results shown in Table 4 and Fig. 4. Furthermore, it allows inclusion of all children into further investigations of the relevance of the migraine versus tension-type-headache distinction. We found the clearest association between the index and aggravation by sports, which is in line with the diagnostic criteria, stating that migraine often is aggravated by physical activity whereas tension-type headache is not. For the other variables, the associations were less clear, but lower index values were found for short-lasting episodes and higher values for long-lasting. This could imply that children with migraine are more affected than those with tension-type headache.

Further studies are necessary to explore the usability of such an index and it should be compared to the gold standard for headache classification to assess the potential utility. Hypothetically, if a certain cut point could be established in order to increase knowledge on appropriate treatment strategy, that could be very helpful for the clinician in charge. This seems especially important due to the high variation in management of headaches in this cohort. Given the known risk of medication overuse headache [29, 30] and the known side effects of migraine medications [31], it is important to end up with the most likely diagnosis, so the children in need of relevant medicine will receive it, and those who don’t will not.

### Strengths and limitations

The basic limitation of the study was the lack of information on some of the criteria of the ICHD classification, possibly implying an imperfect classification. In contrast, we had information on many other aspects of interest in children suffering from headache. However, we are aware that there are weaknesses associated with using questionnaire data. It can never be as nuanced as an interview, where it would be possible to get more nuanced answers and thereby minimize the amount of non-classifiable headaches. The insights obtainable from this type of epidemiological research may, however, also inform the clinical setting characterized by one-to-one interviews.

This population is a selected sample of parents and children willing to participate in a trial and to receive chiropractic treatment and is therefore not directly generalizable. However, there were no fees involved and therefore participation was an option for everyone despite income.

This study included children with recurrent or chronic headaches. However, children with less serious headaches also requires attention since early prevention could decrease the prevalence of more serious cases and thereby have a huge impact on future health and quality of life. Since headaches in general are very common, the full picture is better achieved if we not only consider the most bothersome headaches [32].

## Conclusions

This study describes a selected cohort of children with recurrent headaches and highlights that they are quite severely affected, has a rather large intake of medication and that there is a great variation in management.

Although only questionnaire data could be used, requiring a modification of the ICHD criteria, it was feasible to distinguish between migraine and tension-type headaches in nearly half of the children. The large group of non-classifiable headaches might include probable migraines and probable tension-type headaches, as well as mixed, cervicogenic and medication overuse headaches. A migraine-tension-type-index can be generated allowing to include all children in the diagnostic assessment and may be used for further management decisions.

Given the risk of lifelong trajectories of recurrent headaches, good diagnostic tools are essential to explore the options of best possible care and management as early as possible.

## Data Availability

Relevant anonymized data are available from the corresponding author on reasonable request.

## Appendix 2

See Table 5.

**Table 5:** List of variables

Variable*	Question	Response format
*Headache characteristics*		
Frequency of headache	How often do you have a headache?	1–2 days a week, 3–5 days a week, Almost every day
Duration of episode	How long does your headache last?	Less than 2 h, 2 h up to half a day, The whole day, All day and all night. Dichotomized into ‘Less than 2 h’ and ‘2 h or more’
Typical location	Where is your headache most commonly located?	Whole head, Backside of the head, One side of the head, The neck, The forehead, Behind one eye, Varies a lot
Co-occurring symptoms	Do you have other symptoms with your headache?	Nausea, Vomiting, Light sensitivity, Sound sensitivity
Aggravation by sports	Does sport give you a headache?	Yes, no
Typical pain intensity	Pick the number on a 0–10 pain scale that describes your most common headache	0–10 (numerical rating scale; no pain to worst imaginable pain)
*Baseline information*		
Age		7–14
Sex		Male, female
Height		Height in cm (mean, 10^th^ and 90^th^ percentile)
Weight		Weight in kg (mean, 10^th^ and 90^th^ percentile)
Overweight♣		Yes, no
Obese♣		Yes, no
Cervical dysfunction**	Variable generated on the basis on cervical joint dysfunctions found on examination	Yes, no
Decreased cervical motion**	Found on examination	Yes, no
Thoracic dysfunction**	Variable generated on the basis on thoracic joint dysfunctions found on examination	Yes, no
Lumbar dysfunction**	Variable generated on the basis on lumbar joint dysfunctions found on examination	Yes, no
Pelvic dysfunction**	Variable generated on the basis on pelvic joint dysfunctions found on examination	Yes, no
Temperomandibular dysfunction**	Variable generated on the basis on temperomandibular joint dysfunctions found on examination	Yes, no
Duration of headache	How long have you suffered from headache?	0.5–1 year, 1–3 years, More than 3 years
Typical onset of attack	When does the headache most often begin?	Morning, Late morning, Afternoon, Evening/night, Varies over day and night
Co-occurring symptoms	Do you have other symptoms with your headache?	Dizziness, Stomach pain, Visual disorder, Spots in from of eyes, Numbness in arms, Other
Trigger factors	Does any of these give you a headache?	Neck pain, Back pain, Stress, Sitting, Reading, Computer/tv, Menstruation
Relieving factors	Does any of these relieve your headache?	Lying sown, Sleeping, Eating, drinking, Fresh air, Sports, Medicine
Neck pain last year	Have you had neck pain within the last year?	Yes, no
Back pain last year	Have you had back pain within the last year?	Yes, no
Dental braces	Do you wear braces on your teeth?	Yes, no
Non-prescriptive medicine	How often do you do you take non-prescription medication for headache?	Never, 1–3 times a month, 1–3 times a week, More than 3 times a week
Prescriptive medicine	Do you take prescription medication for your headache?	Yes, no
Medication other disease	Do you take medication regularly for other diseases?	Yes, no
Examination general practitioner	Were you ever examined by a general practitioner for your headache?	Yes, no
Examination pediatric specialist	Were you ever examined by a pediatric specialist for your headache?	Yes, no
Previous treatment	Did you ever get treatment for your headache?	Yes, no
Treatment	Who gave you the treatment?	General practitioner, pediatrician, physiotherapist, chiropractor, other (reflexology, massage, other)
Trauma to head/neck without need to see doctor	How many times have you hurt your head and/or neck without seeking a doctor or emergency room?	Never, 1–3 times, More than 3 times
Trauma to head/neck with need to see doctor	How many times have you hurt your head and/or neck and been seen by doctor or gone to the emergency room?	Never, 1–3 times, More than 3 times
Concussion	Have you ever had a concussion?	Yes, no
Allergies	Do you have any allergies (pollen, food, perfume, colourants, animals, dust mite, smoke, other)?	Yes, no
Stomach pain	Do you have stomach pain?	No, Rarely (1–5 times a year), Sometimes (6–12 times a year), Often (more than once a month)
Smoking in the home	Does anybody smoke in your home?	Yes, no
Number of days with sport per week	How many hours per week do you do sports?	0 times, 1–3 times, More than 3 times
Screentime	How many hours per day do you spend on computer/TV/I-pad/mobile phone?	0–1 h, 2–4 h, 5–6 h, More than 6 h
Poor sleep	Do you sleep well?	Yes, no
Menstruation	Do you have menstruation and at what age did it start?	No, Start 8–11 years of age, Start 12–14 years of age
Glasses/contact lenses	Do you use glasses/contact lenses?	Yes, no

*Data from baseline questionnaire if not otherwise indicated. **Data from physical examination

♣ Body mass index (BMI) is widely used as a definition on obesity and overweight, however, in children the BMI changes substantially with increasing age. Therefore, cut off points related to age and sex has been used, utilising data specific reference centiles linked to adult cut off points, to generate two new variables on BMI (overweight and obese) [35]. Children categorized as overweight are not included in the obese category

## Appendix 3

See Table 6.

**Table 6:** Correlation matrix

	Frequency	Intensity	Duration	Length	Medication	Sick days
Frequency	1.0000					
Intensity	− 0.0925	1.0000				
Duration	0.0026	0.0616	1.0000			
Length	0.1090	0.1322	0.0774	1.0000		
Medication	0.0163	0.1381	0.1416	0.0606	1.0000	
Sick days	0.0616	0.2545	0.1891	0.2291	0.1601	1.0000

## Appendix 4

See Table 7.

**Table 7:** Factor loadings of the six variables

Variable	Factor1	Factor2
Frequency	0.0006	0.2953
Intensity	0.4347	− 0.0814
Duration	0.3482	− 0.0159
Medication	0.3039	− 0.0062
Length	0.2942	0.2379

## Appendix 5

See Table 8.

**Table 8:** Pairwise odds-ratios between the four co-occurring symptoms

	Nausea	Vomit	Light sensitivity	Sound sensitivity
Nausea				
Vomit	43.2 *p* < 0.001			
Light sensitivity	2.3 *p* = 0.001	6.4 *p* < 0.001		
Sound sensitivity	2.4 *p* = 0.001	1.8 *p* = 0.06	4.3 *p* < 0.001	
